# Néstor-Guillermo Progeria Syndrome: a biochemical insight into Barrier-to-Autointegration Factor 1, alanine 12 threonine mutation

**DOI:** 10.1186/s12867-014-0027-z

**Published:** 2014-12-12

**Authors:** Nicolas Paquet, Joseph K Box, Nicholas W Ashton, Amila Suraweera, Laura V Croft, Aaron J Urquhart, Emma Bolderson, Shu-Dong Zhang, Kenneth J O’Byrne, Derek J Richard

**Affiliations:** School of Biomedical Science, Institute of Health and Biomedical Innovation at the Translational Research Institute, Queensland University of Technology, Brisbane, QLD Australia; Center for Cancer Research and Cell Biology, School of Medicine, Dentistry and Biomedical Sciences, Queen’s University Belfast, Lisburn Road 97, Belfast, UK

**Keywords:** Progeria, Nuclear envelope, Aging

## Abstract

**Background:**

Premature aging syndromes recapitulate many aspects of natural aging and provide an insight into this phenomenon at a molecular and cellular level. The progeria syndromes appear to cause rapid aging through disruption of normal nuclear structure. Recently, a coding mutation (*c.34G* > *A* [p.A12T]) in the Barrier to Autointegration Factor 1 (*BANF1*) gene was identified as the genetic basis of Néstor-Guillermo Progeria syndrome (NGPS). This mutation was described to cause instability in the BANF1 protein, causing a disruption of the nuclear envelope structure.

**Results:**

Here we demonstrate that the BANF1 A12T protein is indeed correctly folded, stable and that the observed phenotype, is likely due to the disruption of the DNA binding surface of the A12T mutant. We demonstrate, using biochemical assays, that the BANF1 A12T protein is impaired in its ability to bind DNA while its interaction with nuclear envelope proteins is unperturbed. Consistent with this, we demonstrate that ectopic expression of the mutant protein induces the NGPS cellular phenotype, while the protein localizes normally to the nuclear envelope.

**Conclusions:**

Our study clarifies the role of the A12T mutation in NGPS patients, which will be of importance for understanding the development of the disease.

## Background

Aging is a natural process that affects all organisms, although the precise mechanisms of its progression remain poorly understood. As such, human premature aging syndromes, which recapitulate many aspects of natural aging, may allow us to further investigate this phenomenon at the molecular and cellular level. These syndromes largely result from heritable genetic alterations that mainly affect DNA repair proteins, or proteins associated with the nuclear periphery [1,2]. For instance, one category of premature aging syndromes, known as Human Progeroid syndromes (or laminopathies), are caused by mutations in nuclear lamins or other proteins of the nuclear envelope. As a result, cells from these patients are characterized by nuclear envelope dysfunction, altered nuclear activity, impaired structural dynamics and aberrant cell signaling [3]. These irregularities may manifest in premature aging, as well as conditions such as neuropathy.

Recently, two unrelated patients who exhibited several Hutchinson-Gilford Progeria syndrome-like phenotypes were described [4,5]. These patients, however, did not present with many of the symptoms common to those with known human Progeroid syndromes. For instance, neither patient showed signs of ischemia or atherosclerosis, both fundamental phenotypes of Hutchinson-Gilford Progeria syndrome. Cognitive function was also identified as normal in both patients. Moreover, the age of the patients studied was not consistent with the current understanding of known human Progeroid syndromes, with these patients being much older than the average life span of progeroid patients. This condition was named Néstor–Guillermo Progeria Syndrome (NGPS). Whole-genome and exome sequencing of both affected patients identified them as homozygous for a mutation in the Barrier-to-Autointegration Factor 1 (*BAF1* or *BANF1*) gene [5]. This mutation (*c.34G* > *A* [p.Ala12Thr]) results in the expression of a BANF1 protein where alanine 12 is mutated to a threonine residue.

BANF1 encodes a protein consisting of 89 amino acid residues with a molecular weight of approximately 10 kDa [6]. Nuclear magnetic resonance and crystallographic studies have determined that BANF1 may form a homodimer, which is the active state required to bind chromatin [7,8]. During G1, S and G2 phases of the cell cycle, BANF1 is known to predominantly associate with the nuclear envelope, where it interacts with the Lamina associated polypeptides Emerin-MAN1 (LEM) domain of the nuclear scaffold proteins MAN1 [9], Emerin [10] and LAP2 [11,12]. Here, BANF1 regulates organization of the chromatin structure at the nuclear envelope by condensing DNA via a looping mechanism [13], as well as by binding histone H3 and histone linker H1.1 [14]. The interaction between BANF1, chromatin and protein from the lamina is tightly regulated, allowing proper nuclear assembly and chromatin organization during cell cycle progression [15,16]. In addition to these roles, BANF1 has been proposed to regulate the transcription of specific genes [17], to suppress the integration of retroviruses within the genome [6,18,19], and to regulate specific developmental signals [15,20].

Despite the available data on BANF1 biology, the contribution of the A12T mutation to the development of Nestor-Guillermo Progeria Syndrome is poorly understood. In their original study of the disease, Puente *et al*. observed reduced levels of mutant BANF1 in patient fibroblasts, and thus proposed that the mutation may affect protein stability [5]. It was therefore assumed that the observed phenotype of these patients was due to physiologically low levels of BANF1 [5]. In the present study, we aimed to further decipher the role of the BANF1 A12T mutation in the molecular processes leading to the development of the disease. To do so, we used a series of biochemical and molecular tools to understand the defect resulting from this genetic mutation.

## Results

**Figure 1:**
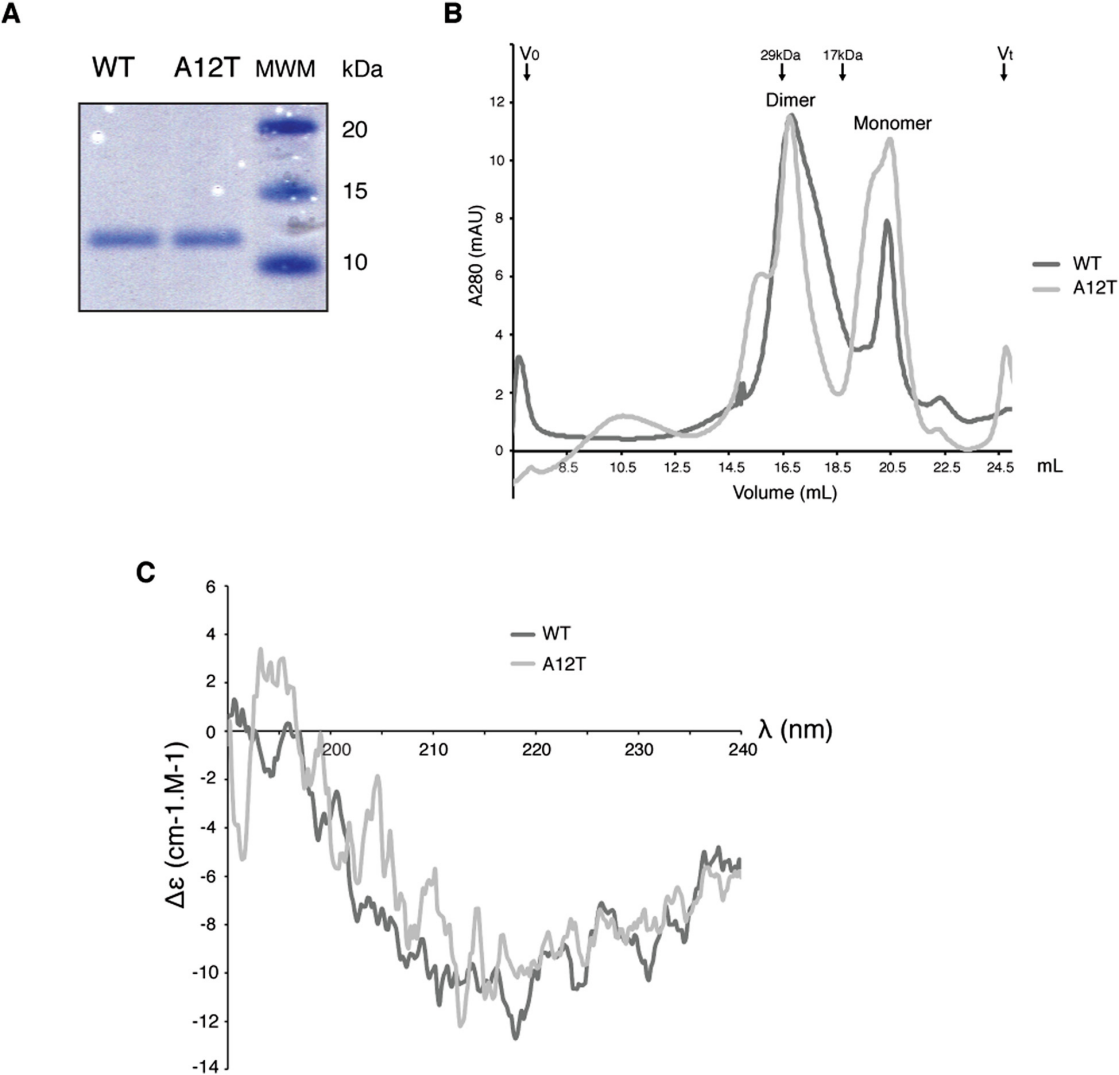
**Characterization of WT and A12T Barrier to Autointegration 1 proteins. (A)**: Coomassie blue staining of 500 ng of recombinant wild type and A12T HexaHis-tagged BANF1 run on a Nu-Page gel. **(B)**: Gel filtration chromatography of wild type and A12T mutant BANF1 proteins. Absorbance at 280 nm was plotted against the elution volume. Both proteins eluted in two predominant peaks, which are consistent with dimeric and monomeric BANF1. Vo indicates the void volume of the column and Vt indicates the termination volume. Arrows indicate the elution volume of the protein standards carbonic anhydrase (29 kDa) and Myoglobin (17 kDa), which were used to calibrate the column prior to BANF1 filtration. **(C)**: A12T BANF1 secondary structure is not modified when compared to the WT. CD spectroscopy of wild-type and A12T BANF1 protein. The mean residue ellipticity Δε of the indicated protein is plotted against the wavelength, and is the results of 3 independent measurements. Δε is in cm^-1^.M^-1^.

In order to characterize the effect of the A12T mutation on the BANF1 protein and NGPS phenotype, we initially purified recombinant His-tagged BANF1 wild type (WT) and A12T proteins from *E. coli* using a protocol adapted from Harris *et al*. [19], although unlike Harris *et al*, we retained the N-terminal His-tag. As previously described, recombinant WT BANF1 was found in inclusion bodies (as was the A12T mutant), indicating aggregation of the protein in a higher-ordered complex, and thus necessitating denaturation and subsequent refolding of the protein during purification. Using this method, both wild type and A12T BANF1 displayed similar purification characteristics (Figure 1A). BANF1 forms a stable dimer in solution with a dimerization interface formed by helix α3, the C-terminus of α5 and part of the loop linking α1 to the helical turn [7]. Although alanine 12 is not located at the dimerization interface of BANF1, we experimentally tested whether A12T mutation may influence this interface indirectly. To do this, we detected both mutant and wild type protein as they eluted from a size exclusion column (Figure 1B). Here, both wild type and A12T recombinant BANF1 eluted with a profile consistent with a mixture of monomeric and dimeric BANF1, supporting that this mutation does not disrupt the dimerization of the protein *in vitro*.

As alanine 12 of BANF1 is positioned in a loop immediately following helix α1, we hypothesized that mutation of this residue to a bulky β-branched threonine could influence the structure of the BANF1 protein. To assess this, we analyzed recombinant WT and A12T BANF1 by circular dichroism (CD), a standard biophysical technique used to study the secondary structure content of proteins in solution. CD spectra of both wild type and A12T BANF1 showed typical α-helical profiles, with minima in the near far- UV at a wavelength of 220 nm (Figure 1C). By this method we detected a spectrum for wild type BANF1 that was consistent with published data [19]. Furthermore, overlay of the A12T BANF1 spectrum indicated the secondary structure of the protein was not affected as a result of mutation.

**Figure 2:**
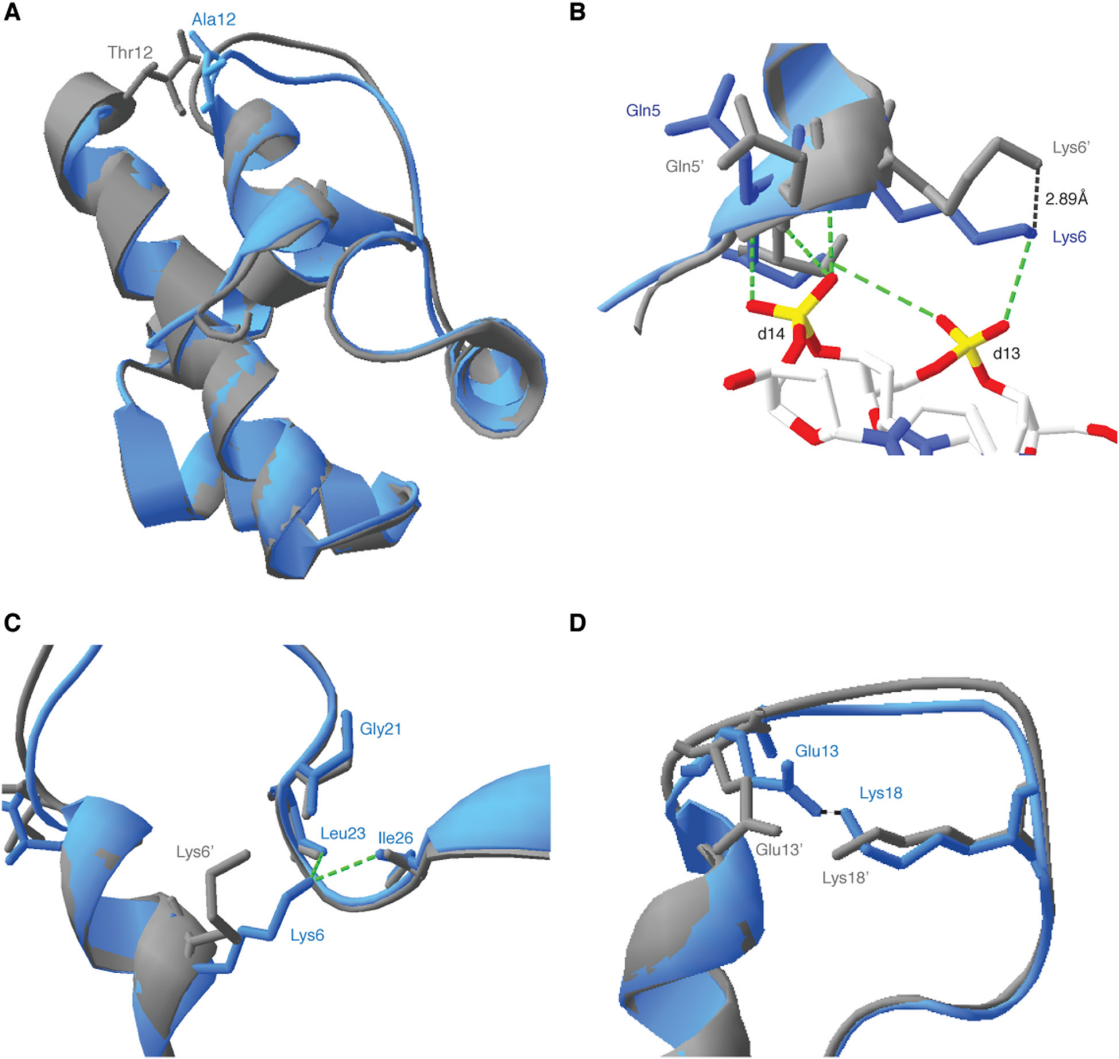
**Structural model of the BANF1 A12T protein. (A-** **D)**: BANF A12T modeling predicts fine changes in the position of key amino acids. WT and A12T sequences were used to generate a model using Phyre2 software. The resulting models were fitted on the existing crystal structure of BANF1 bound to DNA (PDB: 2BZF). **(A)**: Superposition of ribbon diagrams of monomeric WT (Blue) and A12T (gray) BANF1, indicating modification of the loop connecting helix α1 and α3. **(B)**: Ribbons diagrams of WT (Blue) and A12T (Gray) BANF1, indicating the hydrogen bonds between Lys6 and Gln5 with DNA nucleotides d13 and d14 DNA (as depicted in PDB: 2BZF). Lys6’ and Gln5’ from BANF1 A12T are seen displaced from their WT counterpart. **(C)**: Ribbon diagrams of WT (Blue) and A12T (Gray) BANF1. Schematics indicate that in the WT, Lys6 protrudes into the pocket formed by Gly21, Ile26 and Leu23. Lys6’ sits outside this pocket in the A12T mutant. **(D)**: Ribbon diagrams of WT (Blue) and A12T (Gray) BANF1, showing the salt bridge between Glu13 and Lys18 in the WT. Glu13’ and Lys18’ are displaced in the BANF1 A12T prediction.

To explore whether A12T mutation may alter the BANF1 structure on a smaller scale, we obtained the crystal structure of wild type BANF1 bound to DNA from the Protein Data Bank (PDB ID 2BZF), and used Phyre2 [21] (http://www.sbg.bio.ic.ac.uk/~phyre2) to predict the three-dimensional structure of BANF1 following A12T mutation. Modeling was subsequently confirmed using I-TASSER [22] (http://zhanglab.ccmb.med.umich.edu/I-TASSER/). Consistent with the CD spectra data, Phyre2 model prediction of the A12T mutant did not suggest major modifications to the structure of BANF1, although interestingly did indicate a potential alteration in the position of amino acids essential for DNA binding (Figure 2A). Based on the crystal structure, the N terminus of BANF1 helix α1 is important for contacting DNA and establishing hydrogen bonds between Gln5, Lys6 and the nucleotide phosphates [7]. In our model, we therefore predict that the BANF1 A12T mutation may displace the side chain of Lys6 from its original position (Figure 2B), preventing the formation of hydrogen bonds between this residue and the phosphodiester backbone and thus indicating a possible DNA binding defect. Interestingly, Lys6 is also known to be buried in a pocket formed by the carbonyl groups of Gly21, Ile26 and Leu23 [7]. In our prediction, the Lys6 ε-amino group sits outside this pocket, which is likely to have structural and functional consequences (Figure 2C). In addition, our model also predicts the side chains of Glu13 are displaced, potentially affecting the formation of the salt bridge between this residue and Lys18 [23] (Figure 2D). Both Lys6 and Glu13 are important for the stabilization of helix α1 and the loop connecting α1 and α3, which brings positively charged Lys6 and Arg8 to the DNA binding site.

**Figure 3:**
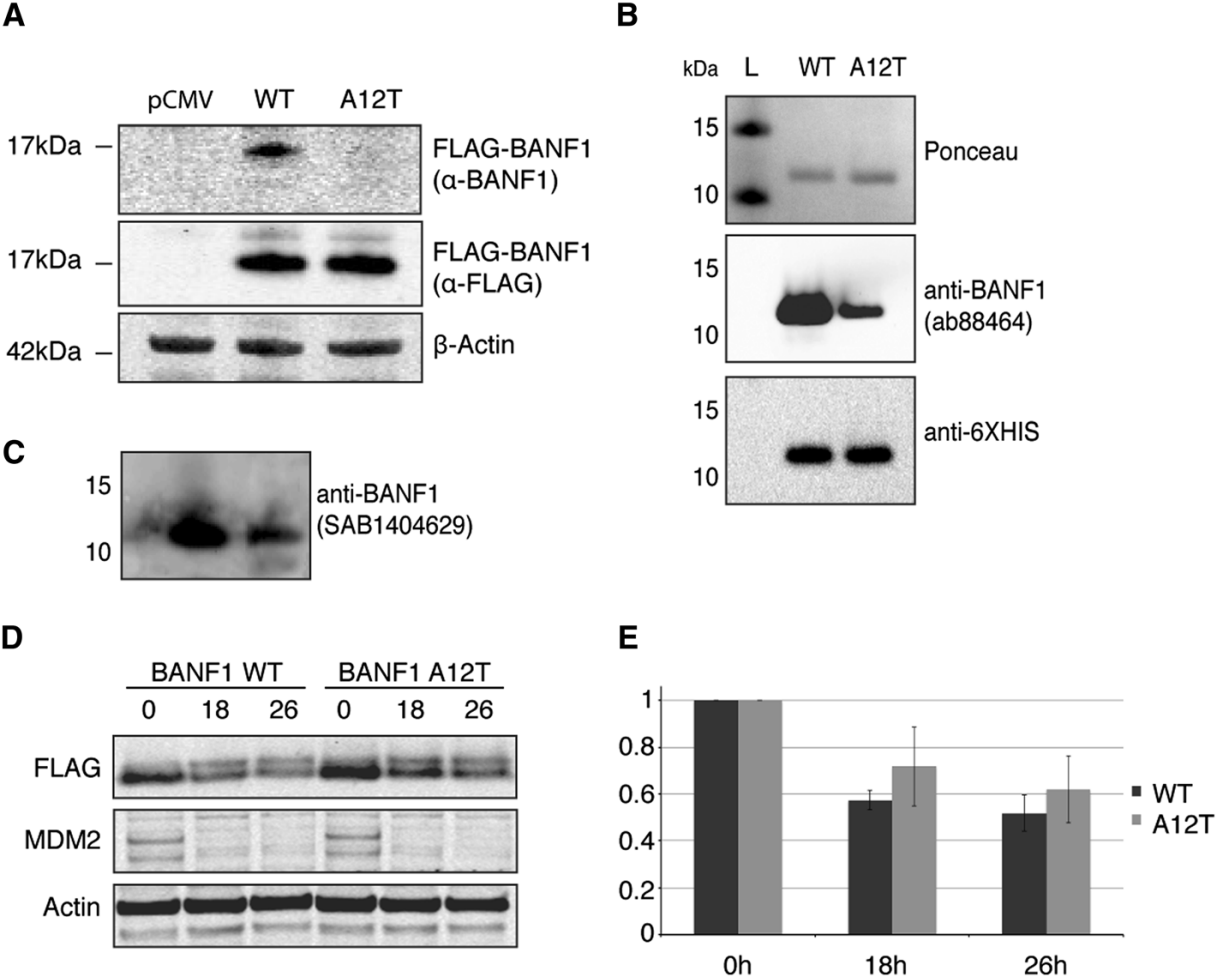
**Alteration of BANF1 A12T antigenicity and stability. (A)**: The anti-BANF1 antibody (Abcam: ab88464) does not recognize exogenous A12T BANF1 in cell lysates. Cell extracts of U2OS cells overexpressing WT or A12T 3x FLAG-tagged BANF1 or an empty vector (pCMV-AN-3DDK) were resolved on a Nu-page gel and immunoblotted using an anti-BANF1 antibody. Membranes were stripped and immunoblotted using an anti-FLAG antibody. A β-actin antibody was used as an internal protein loading control. **(B)**: Recombinant A12T BANF1 has a lower antigenicity than WT BANF1. 500 ng of recombinant HexaHis tagged WT and A12T BANF1 were run on a Nu-page gel and subsequently transferred to a nitrocellulose membrane. The membrane was stained using Ponceau red as a loading control, then blotted with an anti-BANF1 antibody (Abcam ab88464). After visualization, the membrane was stripped and immunoblotted using an anti HexaHis antibody. **(C)**: Recombinant A12T BANF1 has a lower antigenicity than WT BANF1. BANF1 was visualized by blotting with an anti-BANF1 antibody (SAB1404629). **(D)**: Wild type and A12T mutant 3x FLAG BANF1 protein stability. Cells were incubated with cycloheximide (50 μg/ml) for the indicated time periods and cell lysates harvested for western blot analysis. Protein levels were assessed using anti-FLAG antibodies to detect FLAG- tagged BANF1 and anti-actin antibodies for protein loading. MDM2 degradation is shown as a positive control for the cycloheximide treatment. **(E)**: Quantification of **(D)**. Band signal intensity was quantified using ImageJ and standardized against the protein level a t = 0. Error bars represent the standard deviation (SD) from at least three independent experiments.

To understand further the instability previously described for A12T BANF1, we expressed 3x FLAG-tagged WT or A12T BANF1 in HeLa cells from a CMV promoter. Consistent with the observations of Puente *et al*. [5], we observed minimal levels of the A12T mutant protein when compared to the wild type protein, as determined by immunoblotting using the same BANF1 antibody as in their study (Figure 3A). To confirm that we had indeed detected ectopically expressed FLAG tagged BANF1, we stripped the nitrocellulose membrane of the BANF1 antibody and re-probed with an antibody against the FLAG tag (Sigma, F1804 SL11063). Unexpectedly, this indicated equal expression of both the wild type and mutant BANF1.

These data raise the possibility that alanine 12 is required for antibody recognition of BANF1, such that reduced antigenicity may result from A12T mutation. To test this further, 500 ng of recombinant BANF1 WT and A12T was run on a SDS page gel and transferred to a nitrocellulose membrane for immunoblotting (Figure 3B). The loading of equal quantities of proteins was verified by staining of the membrane using Ponceau red. The immunoblot was then probed using an anti-BANF1 antibody (Abcam ab88464). Similar to our findings using overexpressed WT and A12T BANF1 in cells, the BANF1 antibody failed to recognize the A12T recombinant protein to a level comparable to that observed for the wild type protein. To confirm this result, the membrane was stripped of antibody and then re-probed using an anti-HexaHis antibody (Abcam ab1187) to detect the N-terminal His tag of the recombinant BANF1 proteins. Consistent with the Ponceau staining, the anti-HexaHis antibody demonstrated that both proteins were present in similar amounts (Figure 3B). In an attempt to confirm these findings, further immunoblots using a BANF1 antibody supplied by Sigma (SAB1404629) were performed, and again the BANF1 antibody demonstrated a lack of recognition of the mutant protein (Figure 3C). This experiment was repeated several times with proteins from different purification batches and consistently suggested a reduced antigenicity of the mutant protein when anti-BANF1 antibody was used. Interestingly, a similar alteration of antigenicity of BANF1 as a result of epitope mutation has previously been reported [18]. In this study by Lin and Engelman, an anti-BANF1 antibody raised against a peptide encompassing amino acids 4 to 20 did not recognize K6A and K18A BANF1 mutant proteins. These findings may be explained by the consideration that BANF1 is a small and structured protein and that the antigenic regions recognized by the antibody are likely to be composed of only a few amino acids. The mutation of one of these residues, or a residue impacting the structure of this epitope, may therefore interfere with antibody binding.

The observation that A12T BANF1 may be of reduced antigenicity compared to the wild type protein is of importance, as using the same BANF1 antibody, Puente *et al*. detected a lower signal for BANF1 in cells from NGPS patients [5]. The authors then discussed the possibility that the pathology observed is due to a decrease in the stability of BANF1 A12T. In light of their results however, the hypothesis of a reduced half-life for A12T BANF1 may be inadequate. To investigate whether A12T BANF1 is indeed unstable, we investigated whether the half-life of this protein is reduced in cells compared to the wild type. To do this, we transfected HeLa cells with plasmids expressing 3x FLAG-tagged WT or A12T BANF1 and then treated these cells with cycloheximide to block transcription and subsequent protein synthesis. Cells were then harvested 18 and 26 hours post treatment, lysates were separated by SDS-PAGE and proteins transferred to nitrocellulose. Ectopically expressed BANF1 was then detected by Western blot analysis using the anti-FLAG antibody. As shown in Figure 3D-E, similar decay patterns were detected for both WT and A12T BANF1 over the experimental time course (Figures 3D-E). These data demonstrate similar stability of WT and A12T BANF1. To confirm efficacy of our treatment, we also immunoblotted for MDM2, a protein with a known short-life; as expected, loss of protein was observed by our first time point [24]. We were unable to extend our cycloheximide treatment past 26 hours due to cell death. Taken together, our results provide strong evidence that the BANF1 A12T mutant is stable and that the phenotype seen in the NGPS patients is likely to be due to an altered function of BANF1.

**Figure 4:**
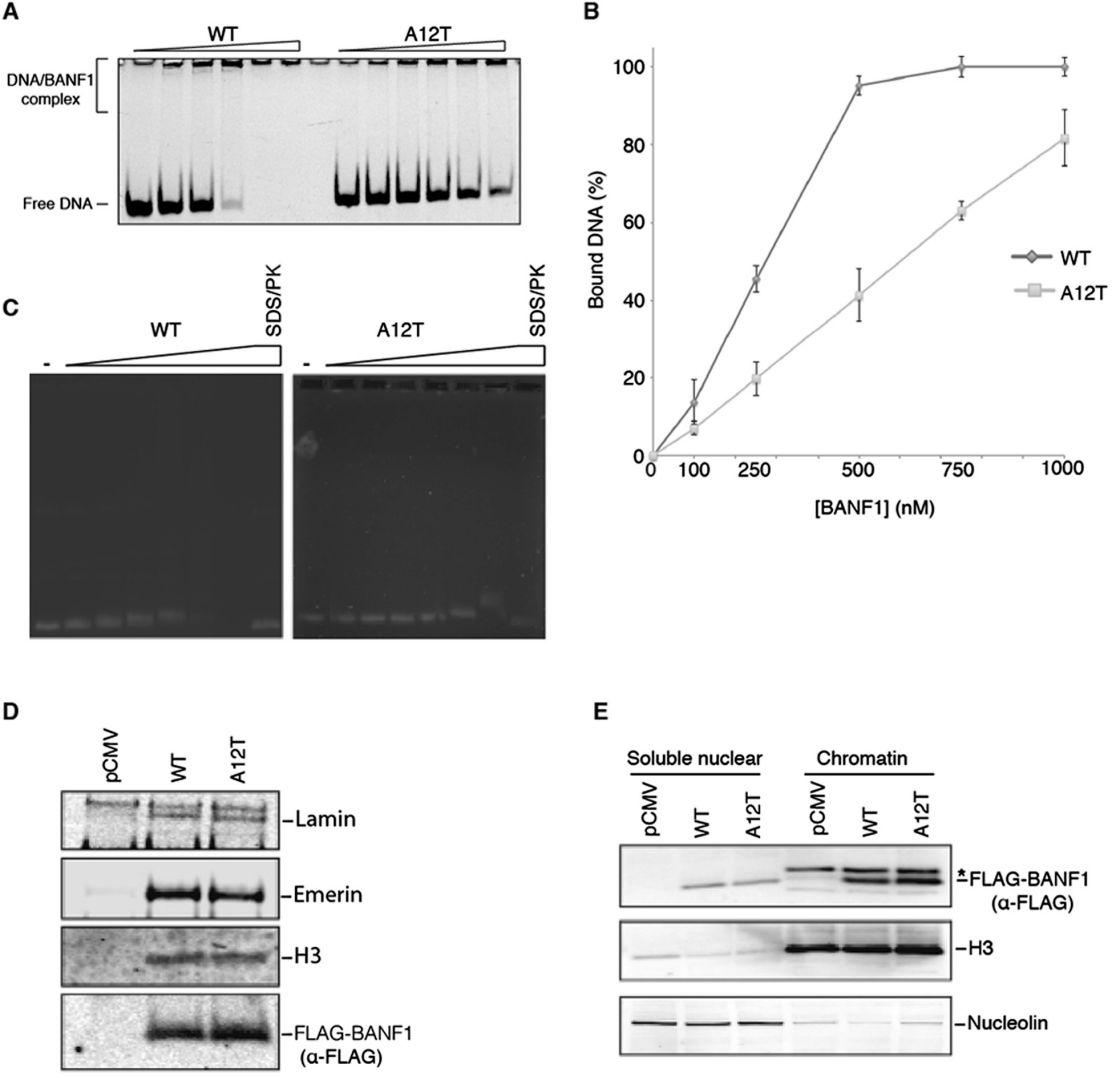
**Alteration of BANF1 A12T DNA binding. (A)**: A12T BANF1 has a reduced affinity for short double-stranded DNA. WT and A12T BANF1 (0, 0.1, 0.25, 0.5, 0.75, 1, μM) was incubated for 30 min at 4°C with 10 nM of dsDNA that was labeled with a 5’ FAM label. The FAM label was visualized using a Starion FLA-9000 image scanner. **(B)**: Quantification of **(A)**. Intensity of the signal was quantified using MultiGauge software (Fujifilm). Error bars represent the standard deviation (SD) from at least three independent experiments. **(C)**: A12T BANF1 has a reduced affinity for long double-stranded DNA. WT and A12T BANF1 (0, 0.1, 0.25, 0.5, 1, 2 and 4 μM) was incubated for 30 min at 4°C with 150 ng of double stranded DNA plasmid. The binding was visualized on an agarose gel following staining with Ethidium bromide. **(D)**: The interaction between WT or A12T BANF1 and known partners was tested by co-immunoprecipitation. HeLa cells were transfected with the indicated vectors and exogenous protein expressed for 24 hours Total protein was then extracted and treated with Benzonase to degrade genomic DNA. M2 magnetic FLAG beads were used to immunoprecipitate 3x FLAG BANF1 and eluent probed using specific antibodies against Lamin, Emerin and Histone H3. **(E)**: A12T BANF1 nuclear distribution is similar to that of WT BANF1. HeLa cells were transiently transfected with the indicated vectors prior to sub-cellular fractionation. Western blotting of fractions was performed using an anti-FLAG antibody (to detect exogenous BANF1), anti-H3 (chromatin fraction loading control) and anti-nucleolin (soluble nuclear loading control). *Bleed-through from antiH3 channel.

As mentioned previously, our structural modeling indicated the presence of a bulky threonine residue, in place of alanine residue 12, might disrupt the DNA binding pocket of BANF1. To investigate this further, we performed DNA mobility shift assays. Escalating concentrations of recombinant purified WT and A12T BANF1 were incubated with a 21 nucleotide double-stranded DNA oligonucleotide. The DNA probe was labeled with a 5’ FAM and the interaction between BANF1 and the DNA observed as retardation in the migration of the complex through a polyacrylamide gel. As previously described, BANF1 WT has a high affinity for double-stranded DNA and bundles DNA in a highly ordered nucleoprotein complex [25]. The kinetics of binding were first studied after incubation for 5 minutes at 37°C, however due to the rate of binding, we were unable to observe intermediate complexes. The reaction was then performed at 4°C for 30 minutes and this allowed us to observe the lower order complexes. Interestingly, the BANF1 A12T mutant exhibited a marked defect in DNA binding compared to the wild type protein (Figure 4A,B). To confirm our observation we conducted DNA mobility shift assay in the same conditions using a 4.5 kb double stranded DNA plasmid. Consistent with our previous observations, A12T BANF1 exhibited a decreased affinity for longer DNA substrates (Figure 4C).

**Figure 5:**
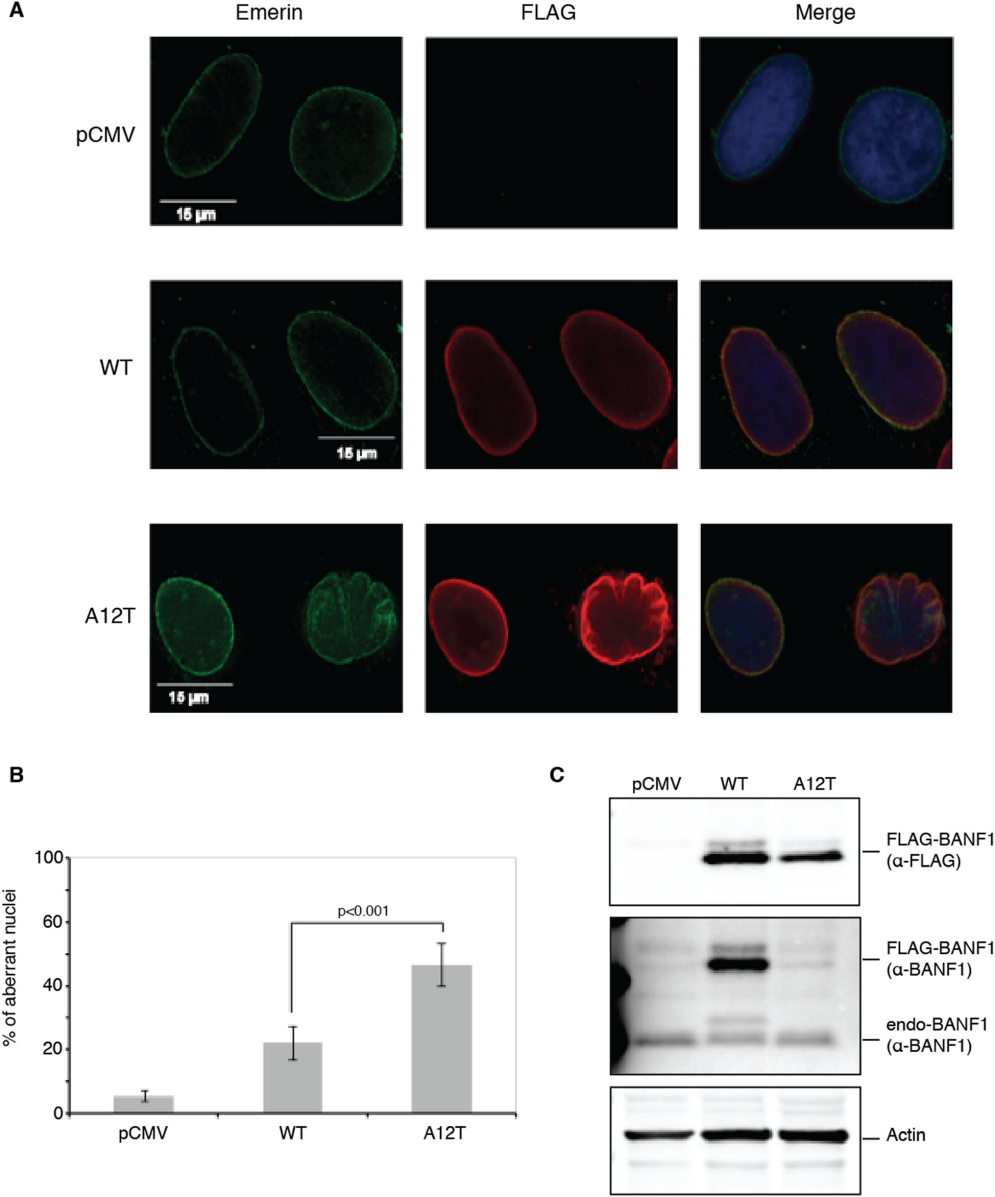
**Nuclear envelope localization of the BANF1 A12T protein. (A)**: A12T BANF1 localization to the nuclear envelope is unaltered from the WT protein. U2OS cells were transfected with constructs expressing WT or A12T 3x FLAG BANF1. Soluble proteins were extracted with detergent and cells permeabilised. BANF1 distribution was analyzed by immunofluorescence using an anti-FLAG antibody and a Deltavision PDV microscope. Aberrant nuclear envelope conformation can be seen in cells expressing A12T BANF1. Emerin visualization is representative of the nuclear membrane. **(B)**: Quantification of the aberrant nuclei seen in the BANF1 A12T overexpression. **(C)**: Immunoblot showing the expression levels of FLAG-tagged BANF1 WT and A12T in comparison to BANF1 endogenous levels.

Several other mutations have been reported to affect the DNA binding properties of BANF1 [19,23,26]. They can be classified based on the severity of the binding defect, as well as their ability to dimerize and to bind Lamina-associated proteins [20,26]. However, to date, none of these mutants have solely impacted DNA binding versus defects in protein binding. To determine if the A12T mutation also affects the association with known nuclear envelope proteins, we performed co-immunoprecipitation experiments. For this, 3x FLAG tagged wild type or A12T BANF1 was expressed and immunoprecipitated in HeLa cells. Immunoblotting was then performed using antibodies against Emerin, Lamin and Histone H3, proteins that have previously been shown to interact with BANF1 at the nuclear envelope [10-12,14]. Interestingly, BANF1 A12T did not display any defects in binding to these proteins (Figure 4D). Interestingly, BANF1 A12T was still present in the chromatin fraction of a subcellular fractionation, consistent with its interaction with histone protein H3 (Figure 4E). Consistent with these observations, immunofluorescence demonstrated that the A12T mutant BANF1 localized normally to the nuclear envelope in U2OS cells. Interestingly, although expression levels of mutant and wild type were equivalent (as determined by immunoblotting with the FLAG antibody), the majority of cells expressing the A12T mutant demonstrated nuclear envelope aberrations consistent with that observed in NGPS patients (Figure 5A,B,C). Together, our data indicates that the A12T mutation of BANF1, found in Nestor-Guillermo Progeria syndrome, causes a disruption of the DNA binding surface, inhibiting its normal interaction with double stranded DNA. The BANF1 A12T mutant however localizes normally to the nuclear envelope, where it interacts with nuclear envelope proteins and chromatin.

## Discussion

The identification of two Progeria individuals with a single point mutation in BANF1 is important for our understanding of these syndromes. Interestingly, Puente *et al*. reported that although mRNA levels of A12T mutant BANF1 were found at similar levels to wild type patients, BANF1 A12T protein was detected at a much lower level. This was originally interpreted as a result of protein instability. In our study, we sought to understand the mechanism through which this may occur. Interestingly, our study suggested that the A12T mutant was not unstable, and that the lower levels of the protein observed were merely an artifact of antigenicity alterations towards the BANF1 antibody, as a result of the A12T mutation. We therefore reasoned that this mutation might affect protein function in other ways that could explain the NGPS phenotype. Our structural modeling of BANF1, predicted that the mutation of alanine 12 to a bulky threonine, could disrupt the BANF1 DNA-binding pocket and thus disrupt the interaction of BANF1 with DNA. We confirmed by EMSA that the A12T BANF1 was indeed perturbed in its ability to bind to DNA, suggesting that the modeling was correct. Further, our modeling and CD spectra analysis suggested that this was the only disruption to the BANF1 structure. Moreover, we found that the A12T mutant, like WT BANF1, localized to the nuclear envelope and interacted with lamin and histone H3. Further studies using nuclear magnetic resonance (NMR) or crystallographic methods would be needed to gain a more detailed visualization of A12T structural modulations.

Although we cannot exclude that the NGPS phenotypes result from an undiscovered role of BANF1, we suggest that the DNA binding deficiency observed in the BANF1 A12T mutant contributes to the cellular phenotypes observed in NGPS. BANF1 has many proposed roles within the cell that requires it to bind to DNA. BANF1 has been shown to simultaneously bind to the nuclear membrane, LEM domain containing protein, LAP2 and DNA *in vitro*, implicating BANF1 as having a crucial role in tethering the chromatin to the nuclear envelope (12). Supporting this, both LAP2 and another LEM-domain, BANF1-interacting protein, Emerin, interact with other major structural components of the nuclear envelope called Lamins. Lamins play a key role in nuclear structure and assembly and mutations in Lamin genes also lead to a human progeria syndrome, Hutchinson–Gilford Progeria Syndrome (HGPS)(2). In HGPS the disruption to the lamina organization, induced by the mutant Lamin proteins leads to areas of weakness around the nuclear envelope, this can cause the chromatin to herniate, pushing out the destabilized membrane. Interestingly, while we were unable to assess NGPS patient cells endogenously expressing mutant BANF1, ectopic expression of the A12T BANF1 did result in herniation of the nuclear envelope, consistent with what has been reported for the patients ((5) Figure 5). It has been proposed that BANF1, through direct binding to LEM proteins and indirect binding to Lamins, may link chromatin to the inner nuclear envelope and this appears to be the case in *C. elegans *(15). In support of this, herein we have shown that mutation of A12T in BANF1 disrupts the DNA binding of BANF1, leading to the disruption of the nuclear envelope.

## Conclusions

Our study now clarifies the role of the BANF1 A12T mutation in NGPS, providing insight into the disease process. Our study has important implications for the treatment of NGPS patients and has provided new mechanistic insights into the function of BANF1 and the nuclear envelope in aging.

## Methods

### Ethics approval

All experimental procedures are approved by the Institutional Biosafety Sub-Committee of the University of Queensland, Brisbane, Australia.

### Plasmids

A pCMV6-AN-3DDK vector containing the BANF1 CDS cloned into the AsiSI and MluI restriction sites was purchased from Origene. The BANF1 CDS was further sub-cloned into pEX-N-His (Origene) using the AsiSI and MluI restriction sites. Enzymes were purchased from New England Biolabs.

A12T mutations were introduced into both BANF1 vectors by site-directed mutagenesis, using the primers A12T F: CCGAGACTTCGTG**A**CAGAGCCCA, and A12T R: CCATGGGCTCTG**T**CACGAAGTCT. PCR was conducted as per: AccuPrime Pfx polymerase (0.02 U.μl^-1^; Life Technologies), 1x AccuPrime Pfx polymerase reaction mix (Life Technologies), primers (0.3 μM) and template (0.8 ng.μl^-1^), then cycling 19x at: 94°C (20 s), 57°C (30 s), 68°C (6 min 30 s). This was followed by Dpn1 (New England Biolabs) digestion (0.8 U.μl^-1^, 2 h, 37°C) and transformation by heat-shock into chemically competent α-select *E. coli* (Bioline). Successful mutagenesis was confirmed by DNA sequencing (Australian Genome Research Facility) using the primer VP1.5: GGACTTTCCAAAATGTCG. Primers were purchased from Sigma-Aldrich.

### BANF1 purification

Plasmids expressing HexaHis-tagged WT or A12T BANF1 were transformed into BL21 (DE3) pLys *E. coli*. Cells were grown at 37°C and protein expression induced with 1 mM IPTG. *E. coli* were harvested 3 h after induction by centrifugation and stored overnight at -80°C. Cell pellet was resuspended in 8 mL of lysis buffer (25 mM HEPES pH 7.5, 150 mM NaCl) per g of cells, and sonicated. Cell lysates were centrifuged for 30 min at 17,00 rpm and the supernatant discarded. The pellet fraction containing HexaHis BANF1 was solubilized in buffer (25 mM HEPES pH 7.5, 150 mM NaCl, 25 mM imidazole) containing 6 M guanidinium chloride, and kept under agitation for 1 h at 4°C. The lysate was then further centrifuged and the clarified supernatant incubated with HIS-Select® *Nickel Affinity* Gel for 2 h at 4°C, under agitation. The affinity gel was extensively washed with the solubilization buffer and the protein was eluted from the beads in buffer K (20 mM KH_2_PO_4_, pH 7.4, 0.5 mM EDTA, 10% glycerol, 0.01% IGEPAL) complemented with 300 mM KCl and 250 mM Imidazole. Eluents were supplemented with 100 mM DTT and incubated for 2 h at 40°C to reduce any remaining disulfide bonds.

Protein was then concentrated on a 10 kDa cut-off *Microsep*™ centrifugal device (Pall corporation) to a volume of 250 μL and loaded on a *Superose 6 10*/*300* GL size exclusion chromatography column (GE healthcare) run with K buffer containing 300 mM KCl. High molecular weight fractions containing BANF1 were discarded and fractions containing monomeric BANF1 at near homogeneity pooled, concentrated and stored at -80°C.

### Protein model

Amino acids sequences from BANF1 WT and A12T were used to generate three-dimensional model with Phyre2 (http://www.sbg.bio.ic.ac.uk/~phyre2). Modeling was subsequently confirmed using I-TASSER (http://zhanglab.ccmb.med.umich.edu/I-TASSER/). Models generated were visualized and analyzed using Swiss PDB viewer [27].

### Cell lines

U20S and HeLa cells were cultured in RPMI 1640 medium (Sigma-Aldrich) containing 10% FCS and maintained in a humidified incubator at 37°C/5% CO_2_.

### Transfection of FLAG-tagged Banf1 constructs

U2OS and HeLa cells were transfected with pCMV6-AN-3DDK, WT or A12T 3x FLAG-tagged BANF1 constructs using Lipofectamine™ 2000 (Invitrogen) as described by the manufacturer. Expression of the 3x FLAG-tagged BANF1 constructs was determined 24 h post-transfection by immunoblotting with an anti-FLAG antibody. Cellular fractionation and immunofluorescence was also carried out 24 hours post transfection.

### Antibodies

Primary antibodies used are as follows: ant-BANF1 (Abcam: ab88464, monoclonal, Sigma: SAB1404629, monoclonal), anti-HexaHis (Abcam ab1187), anti-FLAG M2 (Sigma, F3165), anti-Histone H3 (Cell Signaling, 9715), anti-nucleolin (Cell Signaling, 12247S) and anti-Emerin (Cell Signaling, 5430S). Fluorescent secondary antibodies used are: Donkey anti-Mouse 800 nm (LiCor; IRDye 800CW 926-32212), Donkey anti-Rabbit (LiCor; IRDye 680LT 926-28023) and Alexa Fluor 488 and 594 (Molecular Probes).

### Cycloheximide block

HeLa cells were seeded in 6 cm dishes (300, 000 cells per dish) and the following day transfections were carried out using Lipofectamine 2000 as per the manufacturers instructions using 2 μg plasmid DNA per dish. Cycloheximide (Sigma-Aldrich) was added to the dishes 24 h post transfection at a final concentration of 50 μg/ml and cells were incubated for the indicated amount of time. For the t = 0 h time point, cells were harvested immediately after addition of cycloheximide.

### Cellular fractionation

Cells were separated into cytoplasmic, membrane bound, soluble nuclear, chromatin and cytoskeletal fractions using the Subcellular Protein Fractionation Kit for cultured cells (Thermo Scientific), according to the manufacturer’s instructions. Protein concentrations were estimated using a Bicinchoninic acid assay (Sigma) and subsequently 10 μg of the soluble nuclear and chromatin fractions were separated on a 4-12% SDS-PAGE gel (Invitrogen) and immunoblotted with the indicated antibodies.

### Western blot

Proteins were resolved on 4-12% gradient Nu-PAGE gels (Life Technologies) and transferred to nitrocellulose membrane. Membranes were blocked in 2% v/v fish gelatin (Sigma) in PBS-T for 30 min and incubated with the anti BANF1 antibody diluted in 1% fish gelatin in PBS-T overnight at 4°C. Membranes were washed in PBS-T, incubated with secondary antibodies (LiCor) and scanned on an Odyssey infrared imaging system (LiCor). Where necessary, membranes were stripped using a mild stripping buffer (15 g L^-1^ glycine pH 2.2, 1 g L^-1^ SDS, 1% Tween20) and reprobed with the appropriate antibodies.

### Synthetic DNA substrates

All oligonucleotides were purchased from Integrated DNA Technology (IDT), forward: 5’FAM (carboxyfluorescein) - CTCTCCCTTCGCTCCTTTCCTCT, reverse: AGAGGAAAGGAGCGAAGGGAGAG.

All nucleotides were purified on 12% polyacrylamide, 7 M urea gels prior to further use. For dsDNA annealing, equimolar amounts of corresponding oligonucleotides were mixed in annealing buffer (50 mM Tris pH 7.5, 100 mM NaCl, 10 mM MgCl_2_), heated at 95°C for 10 minutes and slowly cooled to room temperature. The substrates were then purified on native 10% polyacrylamide gels.

Concentrations were determined using the OD_260_ and the molar extinction coefficient of the oligonucleotides (with ε_fluoresceine_ = 13,700 L/mol.cm).

### Electrophoretic mobility shift assay

Reaction were carried out in 10 μL of buffer (10 mM Tris HCl pH 7.0, 20 mM NaCl, 100 ng/mL BSA, 5 mM DTT) with 10 nM of FAM labeled DNA duplex with various concentration of WT or A12T HexaHis BANF1. Proteins and DNA were incubated for 30 min at 4°C. Reactions were resolved on 7% polyacrylamide gels in 0.5x TBE buffer run at 4°C for 90 min at 90 V.

Gels were scanned using a Starion FLA-9000 image scanner (Fujifilm) and quantified using MultiGauge software (Fujifilm).

Long substrate assays were performed in 10 μL of buffer with 150 ng of empty pEX-N-His (Origene) with various concentration of WT or A12T HexaHis BANF1. Proteins and DNA were incubated for 30 min at 4°C. Reactions were resolved on 0.6% agarose gels in 1x TBE running buffer and post stained using Ethidium bromide. Images were taken using Bio-Rad’s *Gel Doc* system.

### Immunofluorescence

U20S cells were seeded the day prior to transfection with the 3x FLAG-tagged BANF1 constructs. Following transfection, the cells were grown for 24 h and were subsequently treated for 5 min on ice with extraction buffer (20 mM Hepes, 20 mM NaCl, 5 mM MgCl_2_, 1 mM ATP, 0.1 mM sodium orthovanadate, 1 mM sodium fluoride, protease inhibitor cocktail (Roche), 0.5% IGEPAL (same chemically as obsolete Nonidet P-40), pH 7.5), to remove the soluble proteins. The cells were then fixed with 4% paraformaldehyde and subsequently permeabilized with 0.2% Triton-X for 5 min and then blocked with 3% BSA for 1 h. Cells were then incubated with the indicated primary antibodies for 1 h hour at RT, washed and counterstained with the corresponding Alexa Fluor conjugated secondary antibodies for 1 h at RT. DNA was counterstained with DAP1 (Sigma, D9564). Images were captured using a DeltaVision deconvolution microscope and the figures were assembled using Adobe Photoshop CS6.
